# Utility and rationale for continuous EEG monitoring: a primer for the general intensivist

**DOI:** 10.1186/s13054-024-04986-0

**Published:** 2024-07-16

**Authors:** Ribal Bitar, Usaamah M. Khan, Eric S. Rosenthal

**Affiliations:** Department of Neurology, Massachusetts General Hospital, 55 Fruit St., Lunder 644, Boston, MA 02114 USA

**Keywords:** Continuous EEG, Seizures, Status epilepticus, Neuroprognostication, Intracranial EEG, Ictal-interictal continuum, Encephalopathy, Traumatic brain injury, Delirium, Periodic discharges, Intensivist

## Abstract

This review offers a comprehensive guide for general intensivists on the utility of continuous EEG (cEEG) monitoring for critically ill patients. Beyond the primary role of EEG in detecting seizures, this review explores its utility in neuroprognostication, monitoring neurological deterioration, assessing treatment responses, and aiding rehabilitation in patients with encephalopathy, coma, or other consciousness disorders. Most seizures and status epilepticus (SE) events in the intensive care unit (ICU) setting are nonconvulsive or subtle, making cEEG essential for identifying these otherwise silent events. Imaging and invasive approaches can add to the diagnosis of seizures for specific populations, given that scalp electrodes may fail to identify seizures that may be detected by depth electrodes or electroradiologic findings. When cEEG identifies SE, the risk of secondary neuronal injury related to the time-intensity “burden” often prompts treatment with anti-seizure medications. Similarly, treatment may be administered for seizure-spectrum activity, such as periodic discharges or lateralized rhythmic delta slowing on the ictal-interictal continuum (IIC), even when frank seizures are not evident on the scalp. In this setting, cEEG is utilized empirically to monitor treatment response. Separately, cEEG has other versatile uses for neurotelemetry, including identifying the level of sedation or consciousness. Specific conditions such as sepsis, traumatic brain injury, subarachnoid hemorrhage, and cardiac arrest may each be associated with a unique application of cEEG; for example, predicting impending events of delayed cerebral ischemia, a feared complication in the first two weeks after subarachnoid hemorrhage. After brief training, non-neurophysiologists can learn to interpret quantitative EEG trends that summarize elements of EEG activity, enhancing clinical responsiveness in collaboration with clinical neurophysiologists. Intensivists and other healthcare professionals also play crucial roles in facilitating timely cEEG setup, preventing electrode-related skin injuries, and maintaining patient mobility during monitoring.

## Introduction/background

Continuous EEG (cEEG) is widely used in the intensive care setting due to the high prevalence of seizures among critically ill patients [1–4]. Most seizures in these settings are nonconvulsive, often identified only through electrographic patterns or as “electroclinical” seizures with subtle motor or behavioral changes. Beyond seizure detection, cEEG serves various purposes, including monitoring sedation, diagnosing disorders of consciousness, aiding neuroprognostication, and identifying impending brain tissue ischemia [5–9].

Despite its broad utility, the implementation of cEEG remains inconsistent across healthcare settings. A Canadian multicenter observational study involving medical, surgical, trauma, and neurological ICUs revealed that out of 375 screened patients, 34% met the criteria for cEEG monitoring recommended by the European Society of Intensive Care Medicine. Yet, 63% of those patients did not receive EEG monitoring despite their eligibility [8, 10]. This included 20% of status epilepticus (SE) patients without return to baseline, 67% of intracerebral hemorrhage patients with altered consciousness, and 100% of patients undergoing targeted temperature management after cardiac arrest, among others [10]. This inconsistency, however, may be seen as an opportunity. With appropriate training, intensivists can achieve advanced proficiency in interpreting findings from EEG recordings and quantitative EEG panels [11]. This point-of-care interpretation is likely to improve the timing and appropriateness of referral to neurology and neurophysiology consultants. Together, this framework may optimize collaborative management, including timely diagnosis, effective monitoring of interventions, and meaningful neuroprognostication during cEEG monitoring, all while ensuring patient safety, mobility, and stewardship of resources.

This review aims to outline the diverse applications of EEG in critically ill patients, focusing on the role of cEEG in detecting seizures and SE, monitoring treatment response, neuroprognostication, and broader applications among patients without evident seizure activity. We provide a guide to the optimal timing and duration of EEG monitoring, the benefits of intracranial EEG for enhancing seizure detection, the impacts of ICU medications on EEG activity, and the importance of multidisciplinary care in ensuring safe and effective monitoring. This comprehensive approach aims to highlight how cEEG can be pivotal in improving patient outcomes and the quality of care in intensive care environments.

## Continuous EEG for seizure and status epilepticus detection

### Detecting seizures and status epilepticus in critically ill patients

A seizure represents a transient occurrence of signs and/or symptoms due to abnormal excessive or synchronous neuronal activity in the brain, manifesting as changes in behavior, movements, and consciousness [12, 13]. Status epilepticus, a more severe form, is defined by the International League Against Epilepsy as 'a condition resulting either from the failure of mechanisms responsible for terminating seizures or from the initiation of mechanisms that lead to abnormally prolonged seizures' (after a context-specific duration, t_1_). This condition can result in long-term neurological injury or death if it extends beyond a critical duration (t_2_) [14]. This operational and contextual definition allows for applying empirical evidence from different seizure types to estimate the duration after which seizures become self-sustaining (t_1_) and the duration after which seizures may cause neuronal injury (t_2_). Understanding these times is important, given that SE is a neurological emergency that requires time-sensitive interventions due to its high mortality and risk of secondary neuronal injury [15].

Convulsive Status Epilepticus is characterized by continuous, prolonged seizure activity with prominent motor symptoms lasting at least 5 min (t_1_ = 5 min). It is likely to cause long-term secondary neuronal damage if persistent for more than 30 min (t_2_ = 30 min) [14, 16]. Convulsive activity refers to episodes of excessive abnormal muscle contractions, bilateral when generalized, which may be sustained or interrupted [17]. Because generalized convulsive SE carries a high mortality risk of up to 20% [18], early recognition warrants prompt intervention [19].

Nonconvulsive status epilepticus refers to ongoing focal or generalized seizure activity without the prominent motor symptoms seen in convulsive SE. Patients may have clinical manifestations that are nonconvulsive, ranging from mild confusion to aphasia or coma [20]. Formal diagnosis of nonconvulsive SE requires at least 10 min of ongoing seizure activity (t_1_ = 10 min), and it is speculated that long-term sequelae ensue at a t_2_ of approximately 60 min [16, 21]. Given the lack of clear semiology, EEG is necessary to support the diagnosis of nonconvulsive SE by identifying electrographic seizure activity of sufficient duration to constitute electrographic status epilepticus.

Continuous EEG is the gold standard for identifying electrographic seizures and SE in hospitalized patients [3, 22]. Depending on the duration and type of EEG monitoring, and the specific ICU populations studied, the incidence of seizures ranges between 3.3 and 34% [23, 24]. This variability, reflects differences in detection methods as well as etiologies [25]. Early detection is advised to minimize complications and improve outcomes, given that secondary brain injury can accrue from hypermetabolism [1]. Remarkably, even after an apparent clinical response to treatment, convulsive seizures and convulsive SE can transform into nonconvulsive seizures that can be detected via cEEG in 48% of patients, in which more than 14% of those manifest nonconvulsive SE [26].

The EEG criteria for electrographic seizures were developed through the Salzburg criteria [27] and standardized in terminology by the American Clinical Neurophysiology Society (ACNS) [16], which defines repetitive epileptiform discharges as > 2.5-Hz activity for ≥ 10 s or any pattern with definite evolution lasting ≥ 10 s [28]. Evolution means having at least two unequivocal, sequential changes (i.e., in the same direction, not fluctuation) in frequency, morphology, or location [16]. When definitive, electrographic seizures do not require correlating clinical symptoms. However, when EEG activity does not fulfill the criteria for electrographic seizures itself, it may help meet criteria for “electroclinical” seizures or electrographic SE when a clinical correlate (e.g., thumb twitching) is time-locked with an EEG pattern of any duration [16].

To diagnose nonconvulsive SE, electrographic or electroclinical seizures lasting ≥ *10 continuous minutes or a total duration of* ≥ *20% of any 60 min* of the recording need to be identified [16]. EEG activity alone lacking criteria for electrographic seizures or nonconvulsive SE may nevertheless display rhythmic and periodic patterns (RPPs) constituting “possible SE” on the “ictal-interictal continuum” (IIC), which can increase the burden of secondary brain injury, as discussed below, even in the absence of a clinical correlate. Of critical importance, IIC activity as “possible SE” fulfills criteria for definite nonconvulsive SE, when EEG and behavioral improvement occur together upon treatment with antiseizure medication (ASM).

### Early EEG monitoring in emergency departments

Encephalopathy due to a neurological disorder is common in emergency department (ED) patients, with nonconvulsive seizures or SE occurring in approximately 5% of ED patients presenting with encephalopathy [29]. Among these patients, the diagnosis is often delayed; for instance, in a group of 23 patients admitted to the ED and later identified with nonconvulsive SE—defined by a minimum 30-min period of altered behavior and EEG-confirmed epileptic activity without convulsions—10 were only diagnosed after 24 h of hospital admission [30]. Such delays in diagnosis stall the timely initiation of appropriate treatment, consequently increasing morbidity and mortality rates [31].

Additionally, a secondary analysis from the established status epilepticus treatment trial (ESETT) found that only 58% (278/475) of a subgroup of patients had an EEG performed within the first 24 h post-seizure onset, with a median time to EEG recording of 5 h (IQR: 3–10 h) [32]. Emergent EEGs are infrequently performed in the ED, especially during nighttime and on weekends, primarily due to logistical challenges associated with the application of EEG in the ED, such as limited space, availability of technologists, and a lack of expertise in acute EEG interpretation, compounded by generally low clinical suspicion [33].

The practice of clinically diagnosing and managing encephalopathic ED patients based on presumed seizure activity also presents issues, as many of these patients do not ultimately show EEG abnormalities or electrographic seizures [29]. Introducing a concise EEG training module for ED physicians, supplemented with quantitative EEG (qEEG) and rapid EEG monitoring techniques, could significantly enhance seizure detection. This improvement could also reduce unnecessary administration of ASMs in the ED setting [34–36]. Such early EEG evaluations could clarify the diagnosis of epilepsy or other seizure disorders, streamlining diagnostic processes amid uncertainties and promoting timely consultations with neurologists or clinical neurophysiologists. This approach not only aids in the rapid identification and management of seizures but also helps optimize overall patient care in emergency settings.

### EEG patterns correlated with the risk of subsequent seizures

**Fig. 1:**
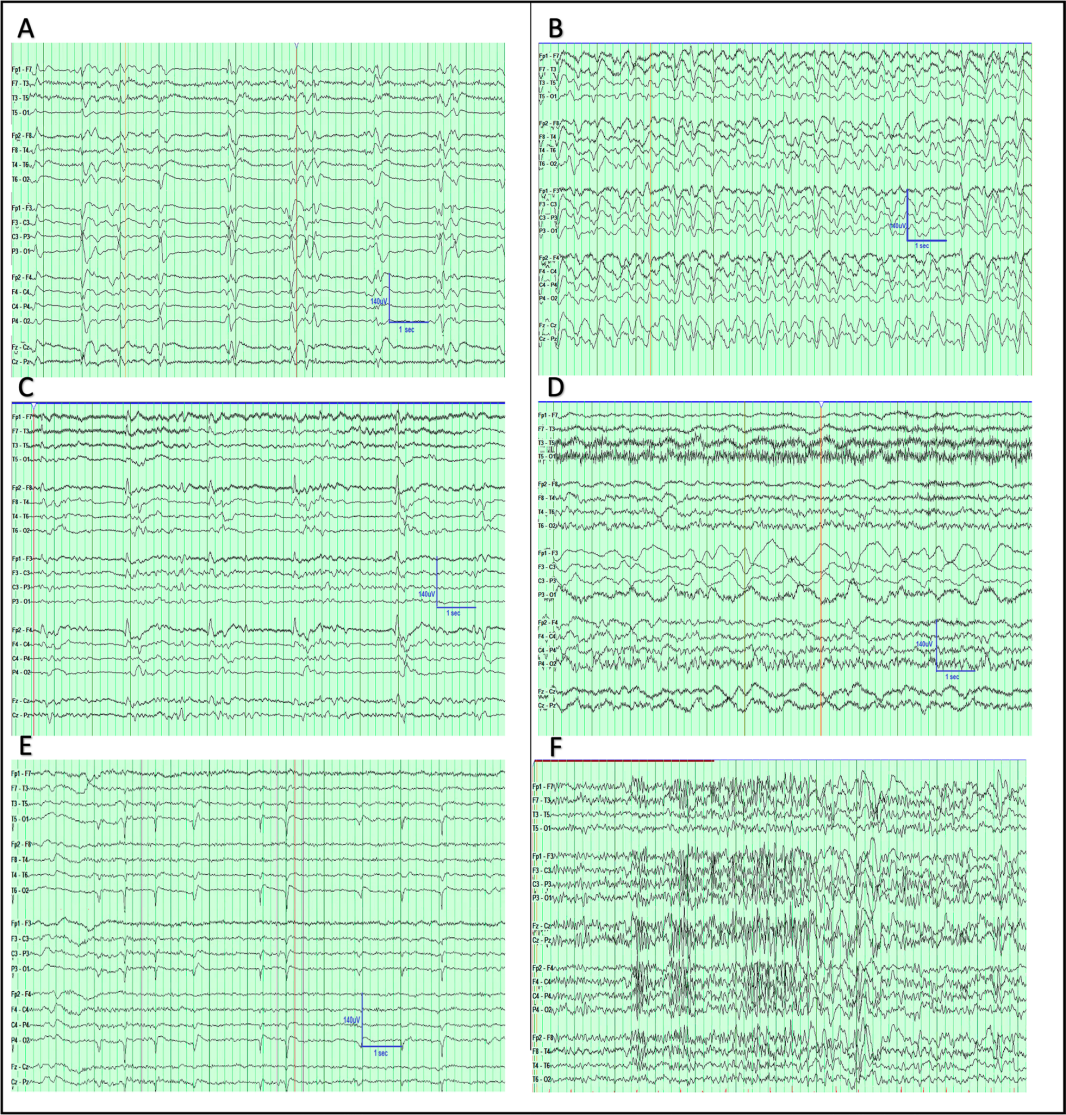
Sample EEGs of various commonly seen rhythmic and periodic patterns. **A **Generalized periodic discharges (GPDs) at roughly 1 Hz Frequency. **B **Generalized rhythmic delta activity + Sharp Activity (GRDA + S) at roughly 2 Hz Frequency. **C** Lateralized periodic discharges (LPDs) Right Frontal predominant at roughly 0.5 Hz Frequency. **D **Lateralized rhythmic delta activity (LRDA), Left Frontal predominant at 1 Hz. Frequency. **E **bilateral occipital independent periodic discharges at bilateral occipital lobes. 0.5–1 Hz Frequency. **F **brief potentially ictal rhythmic discharges (BIRDs) fronto-central predominant

RPP documented on EEG indicate a higher risk of subsequent seizures [37], varying according to its location (Generalized, Lateralized, Bilateral Independent, or Multifocal), pattern (rhythmic delta activity, RDA; periodic discharges, PD; spike-wave; or sharp-wave activity), and secondary modifying features (e.g., periodic discharges with embedded fast activity). Table 1 details the criteria for specific RPP, and Fig. 1 demonstrates sample EEGs illustrating various common RPP instances.

**Table 1:** Description of Electrographic features of commonly seen Rhythmic and periodic discharges. Data was adapted from *Hirsch *et al*.'s J Clinical Neurophys. 2021 and Rodriquez *et al*. JAMA Neurology 2017*

Rhythmic/periodic pattern	Defining features
Generalized Period Discharges (GPDs)	Bilateral or bisynchronous symmetric epileptiform discharges with relatively consistent morphology and duration, with a notable inter-discharge interval between consecutive discharges. Applies to single discharges and not bursts. GPDs can have a restricted field, i.e., Frontally predominant GPDs
Generalized rhythmic delta activity (GRDA)	Bilateral or bisynchronous symmetric relatively uniform repetitive waveforms (no more than 50% variability among each cycle of delta) in the delta frequency (0.5–4 Hz), consistent and without a notable interval between waveforms. GRDA can have a restricted field. i.e., frontally predominant GRDA
Lateralized periodic discharges (LPDs)	Unilateral or bilateral but asymmetric epileptiform discharges with relatively consistent morphology and duration with a notable inter-discharge interval between consecutive discharges. LPDs can be focal, regional, or hemispheric
Lateralized rhythmic delta activity (LRDA)	Unilateral or bilateral but asymmetric relatively uniform repetitive waveforms (no more than 50% variability among each cycle of delta) in the delta frequency (0.5–4 Hz), consistent and without a notable interval between waveforms
Bilateral independent periodic discharged (BIPDs)	The presence of two independent asynchronous lateralized patterns, one in each hemisphere. Each pattern with independently uniform discharges of consistent morphology and duration and notable inter-discharge intervals between consecutive discharges
Stimulus induced rhythmic periodic intermittent discharges (SIRPIDs)	Reproducible patterns of lateralized or generalized intermittent periodic epileptiform discharges induced by an alerting stimulus
Brief potentially ictal rhythmic discharges (BIRDs)	Focal (including lateralized, bilateral and independent, unilateral and independent, or multifocal) or generalized rhythmic activity > 4 Hz (at least 6 waves at a regular rate) lasting ≥ 0.5 to 10 s, that is not consistent with a normal pattern or a benign variant, that has either evolution or morphology similar to epileptiform discharges in a patient

Among RPPs, Lateralized rhythmic delta activity (LRDA) and periodic discharges (PDs), which are generalized (GPDs), lateralized (LPDs), or bilaterally independent (BIPDs), are associated with a greater risk of seizures [37]. However, Generalized rhythmic delta activity (GRDA) is not typically associated with an increased seizure likelihood but may indicate underlying neurological conditions like hydrocephalus, toxic-metabolic dysfunction, subcortical white matter disease, or chronic degenerative disorders [38].

**Fig. 2:**
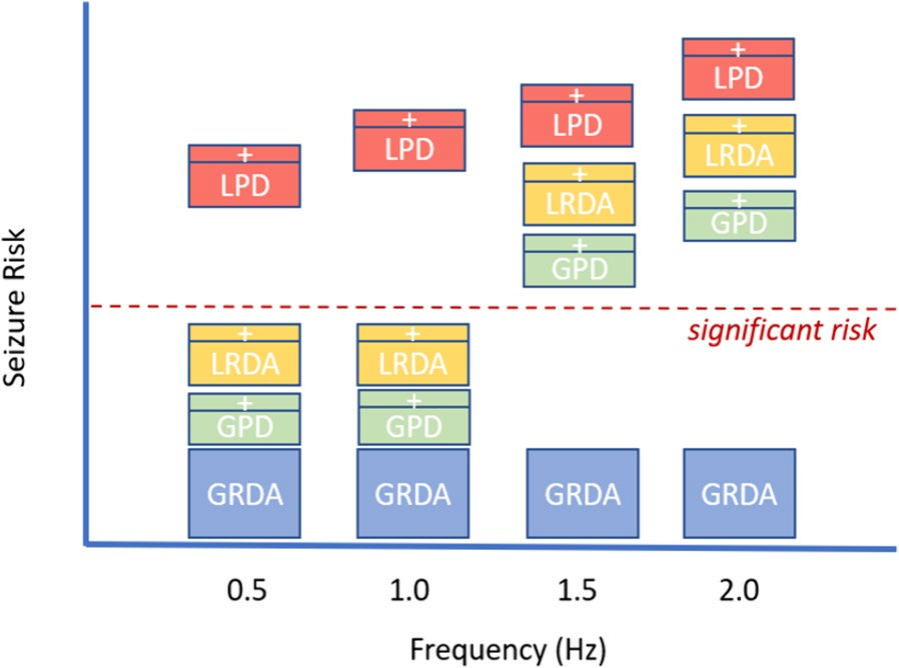
Rhythmic and Periodic Patterns and Seizure Risk Associated with Pattern Frequency. Illustration of variable seizure risk associated with commonly seen rhythmic and periodic patterns on continuous EEG monitoring. The X-axis represents the patterns' frequency, and the Y-axis represents the associated relative seizure risk. Generalized rhythmic delta activity (GRDA), Generalized periodic discharges (GPD), Lateralized rhythmic delta activity (LRDA), Lateralized periodic discharges (LPD), + (plus features [sharp and/or fast activity]). Adapted from Rodriguez Ruiz A, Vlachy J, Lee JW, et al. Association of Periodic and Rhythmic Electroencephalographic Patterns With Seizures in Critically Ill Patients. JAMA Neurol. 2017;74(2):181–188. https://doi.org/10.1001/jamaneurol.2016.4990

Similarly, superimposed sharp or fast activity fulfilling the “modifier” criteria for RDA and PDs is a risk factor for future seizures [37, 39]. Figure 2 details the specific associations between individual EEG patterns and subsequent seizures. Early recognition of RPPs often prompts medical management to attempt to reduce secondary brain injury from this activity, although evidence that such treatment improves outcomes is lacking [40].

### Role of EEG in therapeutic decision-making and outcomes

The Salzburg Criteria and ACNS Critical Care EEG Terminology recognize electroclinical improvement after treatment of a periodic or rhythmic pattern with intravenous ASM as a diagnostic criterion for nonconvulsive SE [16, 27]. This guideline underscores the importance of ASM escalation as a diagnostic trial when IIC patterns are evident. A consensus from experts at the 8th London-Innsbruck Colloquium emphasized the need for specific dosing guidelines during diagnostic challenges or therapeutic intentions [41]. They recommended that the initial dose of the intravenous ASM should be two-thirds to three-quarters of the maximum loading dose used for convulsive SE, with the remaining dose administered if no response or an equivocal response is observed after 15 min. It’s noted that improvements may be delayed due to post-ictal states, potentially lasting several days, especially in patients whose nonconvulsive SE extended beyond 36 h before treatment initiation [42].

Clinical trials and observations show varied responses to ASM or benzodiazepine treatment for triphasic waves—generalized periodic discharges with a triphasic morphology—where 18.9% (10/53) of patients exhibit immediate (< 2 h) clinical and EEG improvements and another 26.7% demonstrated improvement either immediately (< 2 h) or after a delay (> 2 h, but clearly attributed to ASM), related to ASM escalation [43]. However, the use of these ASMs, including benzodiazepines and anesthetics, requires careful management due to potential adverse effects such as respiratory failure.

Continued EEG monitoring and therapy adjustments are often necessary as periodic discharges exceeding 1 Hz can cause focal or regional brain tissue hyperglycosis, and those exceeding 2 Hz may induce brain tissue hypoxia, hyperglycosis, and elevated lactate levels. Such EEG findings may necessitate further escalation of ASM or sedation to mitigate potential brain damage [44–47]. Additionally, observational studies suggest that the risks associated with aggressive therapy for nonconvulsive SE might be offset by the condition’s severity, indicating a need for tailored therapeutic approaches. For instance, the TELSTAR randomized control trial assessed the benefit of ASM escalation among cardiac arrest patients with IIC, electrographic seizures, or electrographic SE, but the subgroup of patients with electrographic SE was small [48].

EEG monitoring can also inform the de-escalation of ASM or anesthetic therapy. For example, EEG can inform readiness for weaning by predicting the potential recurrence of seizures and detecting subclinical seizures that may emerge. When providers commonly target seizure suppression or burst suppression before weaning anesthetic therapy [49], highly epileptiform bursts–bursts with two or more sharp waves or spikes—and burst amplitude are both risk factors for subsequent seizure upon de-escalating anesthetic therapy [50, 51]. Conversely, the re-emergence of functional EEG brain networks (albeit analyzed using a machine learning framework) has been externally validated as a digital EEG biomarker of readiness for anesthetic liberation [52].

### Guidelines and recommendations for effective monitoring

Continuous EEG (cEEG) monitoring is paramount in critically ill patients, offering superior sensitivity over routine or intermittent EEG, which typically entails brief recordings lasting 20–40 min [53–55]. Studies suggest that while brief EEG can be cost-effective and comparable in seizure detection rates as cEEG in certain contexts like post-cardiac arrest patients [56, 57], the prolonged duration of cEEG generally yields better prognostic accuracy for clinical outcome. Notably, cEEG has been associated with reduced in-hospital mortality among mechanically ventilated patients and critically ill hospitalized patients, highlighting its significance in patient management. For instance, two comprehensive cross-sectional studies involving a total of over 7 million patient discharges demonstrated that cEEG monitoring significantly decreased in-hospital mortality compared to routine EEG or no monitoring at all [58, 59]. However, an evaluation of 497 comatose post-cardiac arrest patients referred to the ICU for EEG monitoring (435 with routine EEG and 62 with cEEG) revealed comparable outcomes at three months (mortality at three months was 43.9% in the routine EEG group versus 50.0% for those in the cEEG group; p-value = 0.33) and latency (median of 4 days vs. 5 days in the routine EEG and cEEG groups, respectively, *p*-value = 0.15) [60]. However, these studies did not report on local approaches to the withdrawal of life-sustaining therapy other than institutional guidelines.

Nevertheless, repeated EEG sessions aimed at mimicking continuous monitoring can increase the workload on medical staff without necessarily providing the same level of continuous monitoring efficacy [61].

Rossetti and colleagues highlighted this in a randomized controlled trial comparing routine EEG with cEEG in a diverse group of critically ill patients [62]. They found that while cEEG led to more frequent adjustments in ASMs due to better detection of ictal and interictal features, this did not translate into differences in six-month mortality rates. This underscores the complex role of cEEG in enhancing treatment while also potentially altering prognostic assessments, thus influencing clinical decisions about continuing care.

The efficacy of cEEG is further supported by its ability to guide clinical decisions regarding seizure management. The minimum duration of EEG required for seizure detection is controversial [1] but is best individualized according to the patient's diagnostic and clinical scenario. Monitoring recommendations suggest starting cEEG within one hour of suspected seizure activity [20], continuing for at least 24 h after cessation of electrographic seizures will capture a substantial majority of nonconvulsive seizure activities [53]. Recording for 24 h is thought to detect 88% of nonconvulsive seizures among patients referred for cEEG monitoring while monitoring for 48 h is thought to detect 93% of nonconvulsive seizures [63]. In a study involving 570 patients who underwent cEEG monitoring, seizures were detected in 19% of patients, 56% of whom experienced their first event within one hour of initiating monitoring, and 93% of whom experienced seizures within 48 h [3], although referral bias affected the duration of the recording. Notably, medical conditions associated with a risk of delayed cerebral ischemia, such as SAH, may require longer monitoring to detect seizures, reaching up to 7.3 days to detect 75% of patients with seizures, inf cohorts without referral bias [1, 63].

**Fig. 3.**
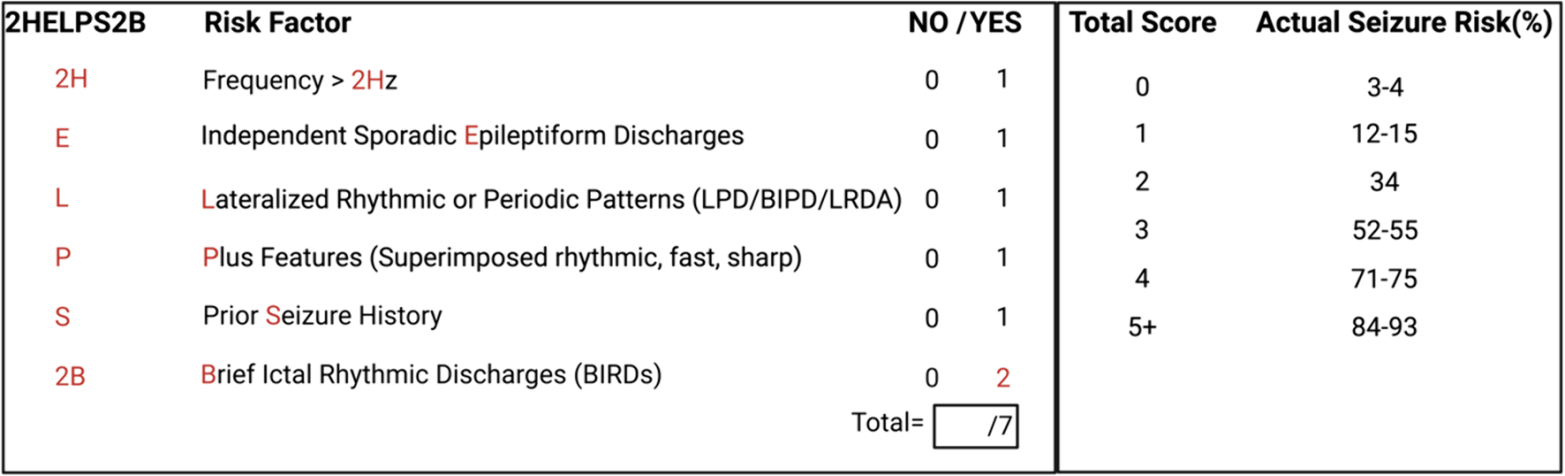
2HELPS2B score. A point system designed to stratify inpatient seizure risk based on 5 electrographic features and one clinical factor (history of seizures). The score was validated to predict seizure risk and guide physicians in determining the minimum required EEG duration. Abbreviations: BIPD, bilateral independent periodic discharge; LPD, lateralized periodic discharge; LRDA, lateralized rhythmic delta activity

However, clinical characteristics are insufficient to determine the necessary duration of EEG. In this setting, the 2HELPS2B score is a tool applied to the first 30–60 min of EEG data that integrates five electrographic factors and one clinical element to guide the risk of subsequent seizures during hospitalization. This tool was developed and validated to predict seizure risk and guide physicians in determining the minimum required EEG duration (Fig. 3) [64–66]. To achieve a false negative rate below 5% risk of seizures, the recommended EEG monitoring duration according to this score is at least 1 h for a score of 0, 12 h for a score of 1, and at least 24 h for a score of 2 or higher [66].

Employing the 2HELPS2B score in real-time at a tertiary care facility reduced the duration of cEEG monitoring without diminishing the seizure detection rate [67]. This quality improvement initiative excluded patients with cardiac arrest, intracranial hypertension, or SE and did not clearly recommend a specific score for triage. However, 59% of the patients had a 2HELPS2B score of 0 before the intervention, and these patients had a 0% seizure rate despite undergoing 33 mean hours of cEEG monitoring. Such an approach of triaging patients based on initial EEG findings may thus enable more precise and targeted long-term neuromonitoring of high-risk patients.

### Comparison of traditional and rapid EEG-approaches

Because qualified EEG staff technologists and interpreting physicians are not always readily available, delays in setting up EEG have inspired the development and implementation of rapid response-EEG tools, which allow for increased EEG monitoring despite the tradeoff in spatial coverage until standard EEG setups can be established [68]. One rapid response-EEG system (Ceribell; Sunnyvale, CA) employs an elastic headband device to record data from 10 electrodes, which can be applied by any healthcare provider with remote review facilitated by physicians. This rapid deployment capability ensures that cEEG monitoring starts without the wait typically associated with traditional setups.

**Fig. 4:**
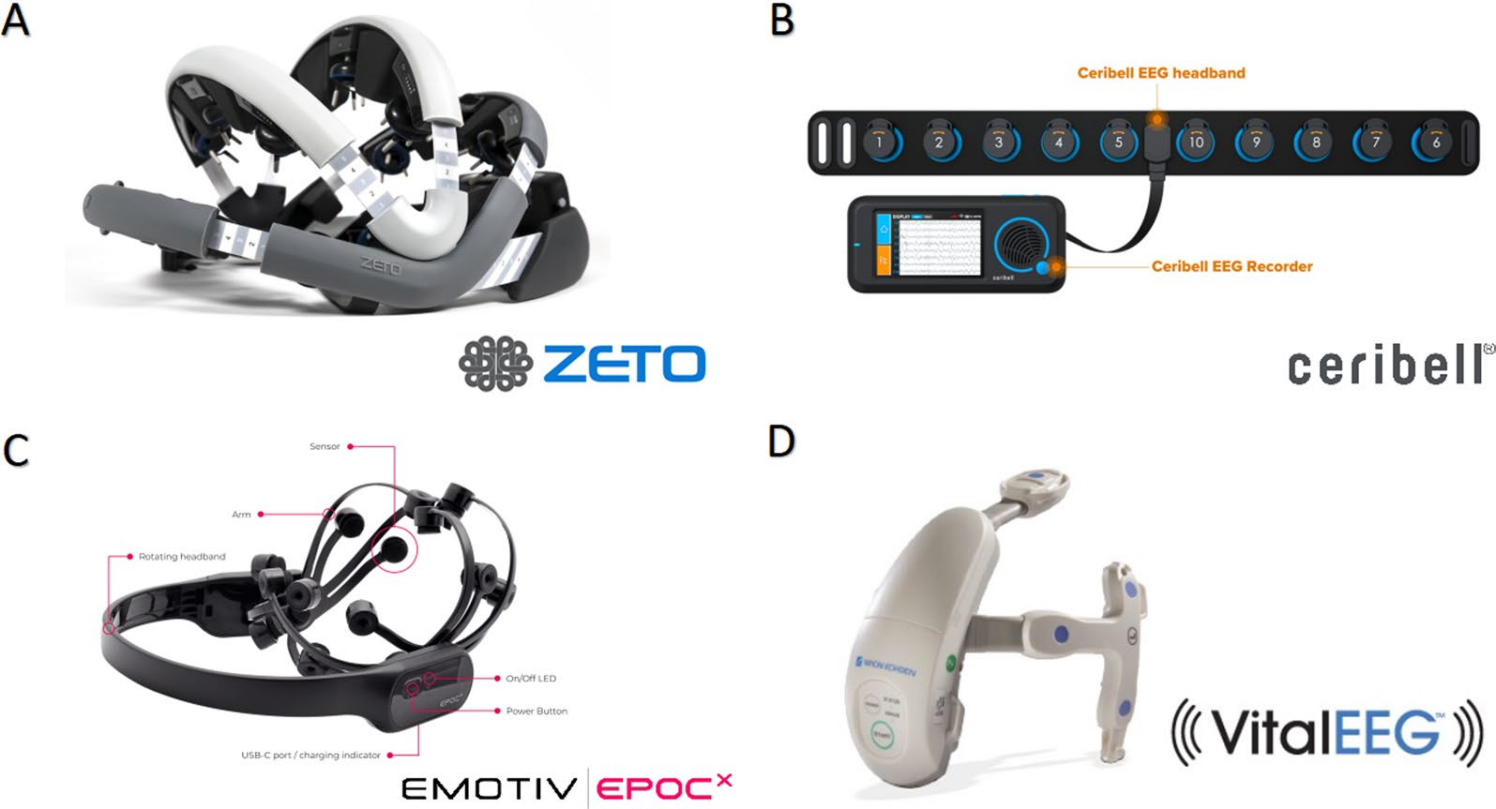
Rapid Response mobile EEGs—**A**: Zeto EEG monitoring device that can be worn like a bike helmet and adjusted according to head size. It has 19 electrodes with A1/A2 reference electrodes and 10–20 system complaints. **B**: Ceribell EEG headband that any healthcare provider can set up rapidly with the pocket-sized Ceribell EEG recorder that provides clinical quality EEG of 10 channel electrodes and on-device EEG. **C**: EMOTIV EPOC^x^ EEG headset is a 14-channel EEG with a 9-axis motion sensor that can detect head movements. It uses Bluetooth technology to wirelessly transmit data to a computer or mobile device to obtain real-time monitoring of brain activity. **D:** VitalEEG™ Wireless EEG Headset is a low channel count that can be rapidly deployed by any ER or ICU nurse and remotely monitored by an EEG technologist or physician. Adapted from: https://zeto-inc.com/device/, https://ceribell.com/, https://www.emotiv.com/epoc-x/, https://us.nihonkohden.com/products/vitaleeg-wireless-eeg-headset/

The efficacy of these rapid EEG systems in clinical decision-making was notably demonstrated in the DECIDE multicenter clinical study [69]. This study assessed the diagnostic and therapeutic outcomes of rapid EEG compared to conventional management relying solely on clinical judgment. Findings revealed improved efficiency and accuracy in physicians' assessments of suspected nonconvulsive SE, with sensitivity for seizure diagnosis increasing from 77.8 to 100.0% and specificity rising from 63.9 to 89% when comparing pre-and post-Rapid-EEG evaluations to the consensus opinion of three epileptologists. Figure 4 displays commonly employed, FDA-cleared, rapid-response EEG devices used for instantaneous bedside recording and rapid seizure detection. Additionally, new tools are becoming available that enable better and more automated identification and classification of seizures and ictal-interictal continuum (IIC) activity using machine learning and artificial intelligence [70–72].

Despite the considerable benefits these point-of-care EEG systems offer, limitations regarding automated software embedded in these systems require consideration. For instance, in a study of 21 patients with coma after cardiac arrest, embedded software for automated detection of seizures [73] failed to detect four individuals (19.0%) experiencing multiple electrographic seizures, 2 of which additionally had electrographic SE within the initial 24-h period of rapid-EEG observation. This highlights the need for cautious interpretation of automated results and, where possible, review by experienced neurophysiologists.

To fully leverage the benefits of rapid EEG, collaboration between neurology, clinical neurophysiology, and intensive care teams is crucial. Such partnerships ensure that rapid EEG findings are accurately interpreted and integrated into patient management strategies. This interdisciplinary approach not only facilitates timely and appropriate interventions for neurological emergencies like nonconvulsive SE but also enhances the overall quality of care through shared expertise and continuous learning [74–76].

## Continuous EEG in specific etiologies

### EEG in prognostication and management of post-cardiac arrest brain injury

Hypoxic-ischemic brain injury after cardiac arrest is the leading cause of mortality and long-term neurological disability among patients who remain unconscious following the restoration of spontaneous circulation [77, 78]. Within the domain of hypoxic-ischemic brain injury, various EEG markers inform prognosis, although caution must be given about self-fulfilling prophecies. Notably, the absence of EEG background reactivity, termed burst suppression, and the presence of epileptiform transients during therapeutic hypothermia have emerged as strong indicators of unfavorable outcomes following cardiac arrest [79–82]. Specifically, the transition towards a continuous normal-voltage EEG background (all activity ≥ 20 μV) within the initial 12–24 h following cardiac arrest is associated with a favorable prognosis [83]. Additionally, timely detection of this normalization correlates with improved prognosis [84]. Other studies, however, have demonstrated a more inconsistent association between the lack of EEG background reactivity and poor outcomes post-cardiac arrest [85–87]. For example, Sivaraju et al. demonstrated that among a cohort of patients admitted with cardiac arrest and undergoing continuous EEG monitoring, the lack of background reactivity on EEG was more common in the poor outcome group; 48/61 (79%) patients vs. 4/28 (14%) patients in the good outcome group (*p* < 0.001), with 86% specificity, 79% sensitivity, and 8% FPR for poor outcome [88]. However, this was not associated with the timing of EEG in relation to the cardiac arrest. Furthermore, it has been established that relying solely on EEG reactivity is inadequate as a predictor of outcomes in post-cardiac arrest patients [86]. Its presence, rather than its absence, enhances the prediction of favorable outcomes with greater sensitivity (95%) compared to predicting poor outcomes [89]. Thus, it is imperative to acknowledge the inherent variability in EEG reactivity interpretation [90], as well as the potential impact of sedative medications administered during therapeutic temperature management on these EEG features [91]. In the absence of the use of high sedative doses, studies suggest that despite sedative infusions in the range of 0·1–0·2 mg/kg per h (midazolam) or 2–3 mg/kg per h (propofol), prognostic accuracy of EEG is higher within the initial 24 h post-cardiac arrest, irrespective of therapeutic temperature management compared to later time points (2–3 days) [88, 92, 93]. While other EEG markers, such as the presence of alpha or theta coma, i.e., monotonous alpha or theta activity, have been suggested to indicate an unfavorable prognosis, they are uncommon, and their predictive utility remains uncertain [94–96]. Additionally, recent research highlights the potential of machine learning algorithms, particularly those integrating functional connectivity features and EEG non-coupling features, to yield high predictive accuracy for identifying poor outcomes (73% sensitivity, 100% specificity, and 0.92 AUC) [97].

**Fig. 5:**
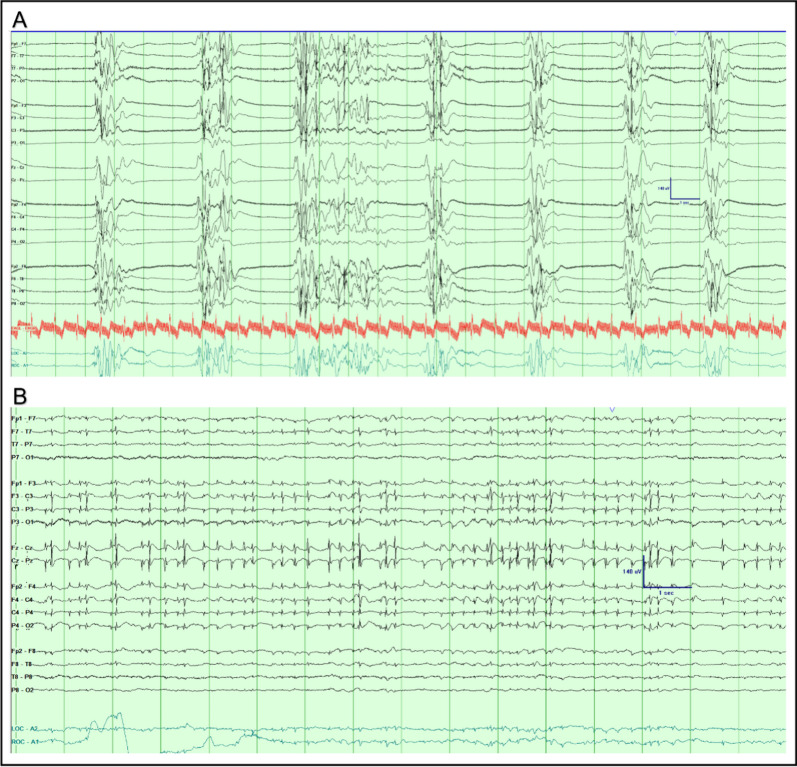
Cortical Myoclonus After Cardiac Arrest: **A** Elmer Pattern 1. Epoch captured on longitudinal bipolar montage with high-pass filter at 1 Hz, low-pass filter at 70 Hz, paper speed of 30 mm/second, sensitivity at 7 uV/mm, and notch filter off. A 65-year-old man with hypoxic-ischemic brain injury following an asystolic cardiac arrest with prolonged time to return of spontaneous circulation. Static periodic highly epileptiform and identical bursts consisting of high amplitude polyspikes captured in lockstep with whole body myoclonus (not shown). **B** Elmer Pattern 2. Epoch captured on longitudinal bipolar montage with high-pass filter at 1 Hz, low-pass filter at 70 Hz, paper speed of 30 mm/second, sensitivity at 7 uV/mm, and notch filter off. A 59-year-old woman with hypoxic-ischemic brain injury following a pulseless electrical activity cardiac arrest with prolonged time to return of spontaneous circulation. Fluctuating midline predominant periodic spikes in lockstep with subtle myoclonus of the face and hands (not shown)

**Fig. 6:**
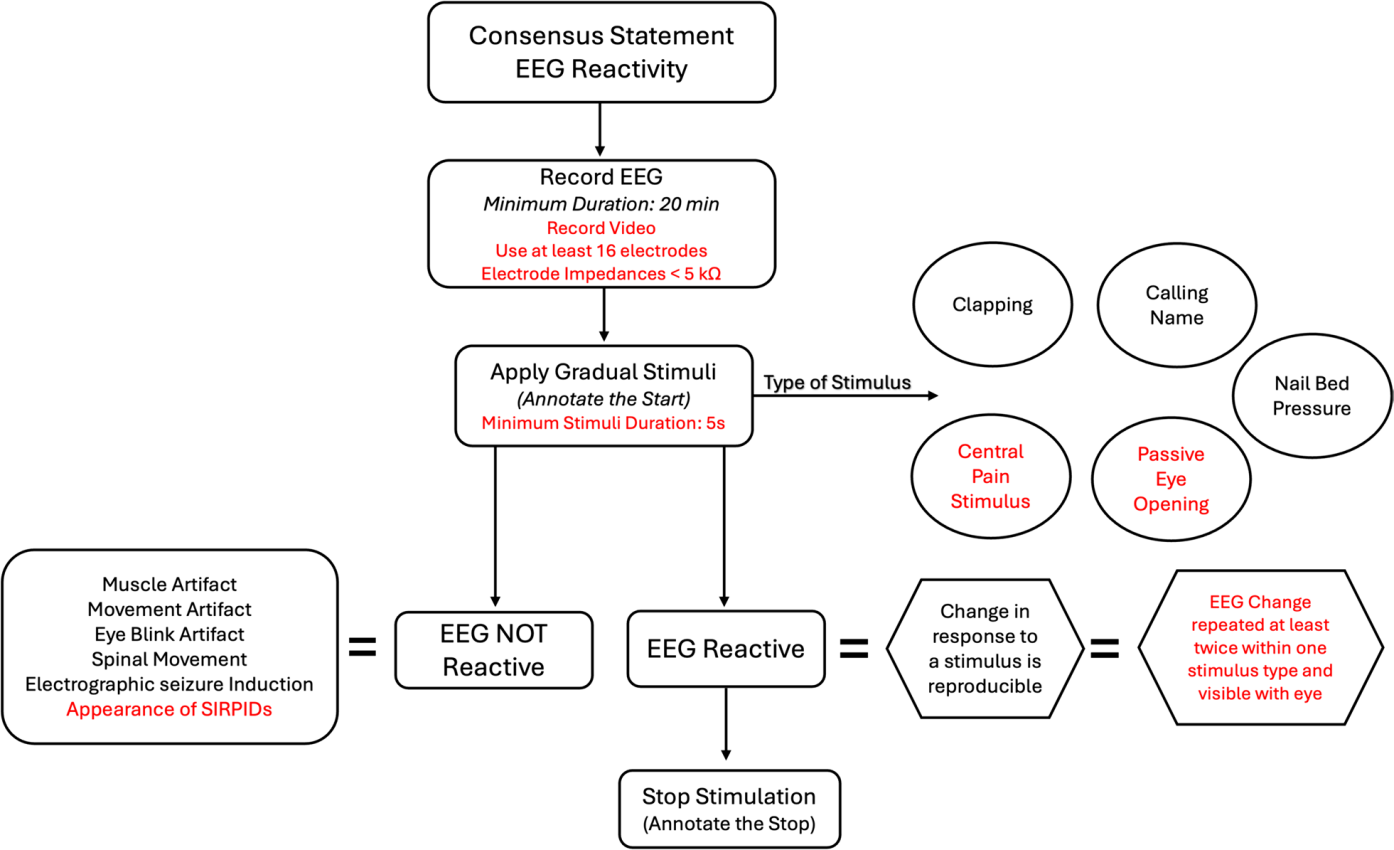
Consensus statement and recommendations for a generalized stimulus protocol for EEG reactivity testing and definition of EEG reactivity in patients after cardiac arrest. The text in black represents the statements derived from the consensus which was defined as ≥ 75% agreement. Text in red represent the set of recommendations that were defined as having a 66–75% agreement. Stimulus induced rhythmic or periodic discharges (SIRPIDs). Adapted from Admiraal MM, van Rootselaar AF, Horn J. International consensus on EEG reactivity testing after cardiac arrest: Towards standardization. Resuscitation. 2018 Oct;131:36–41. https://doi.org/10.1016/j.resuscitation.2018.07.025. Epub 2018 Jul 26. PMID: 30056156

Other than EEG features, studies have consistently demonstrated that seizures and seizure dynamics strongly correlate with outcomes [98–100]. The findings indicate that the presence of seizures, especially nonconvulsive SE, is significantly associated with a worsened prognosis in comatose cardiac arrest survivors. Rittenberger et al. found that among 101 post-cardiac arrest patients, the outcome was poor in the 12% with nonconvulsive SE [98]. Legriel et al. detailed that among 106 comatose cardiac-arrest survivors, postanoxic status epilepticus (PSE) was diagnosed in 33 (31%) patients with a strong, independent correlation with poor outcome [99]. Additionally, a post-hoc analysis of a large, randomized trial with strict criteria for withdrawal of life-sustaining therapy (n = 939) identified an association between early or late seizures and poor outcomes [100]. Myoclonic seizures were the most common and most predictive of poor outcomes. Post-arrest myoclonus has diverse prognostic implications, depending on its source (cortical or subcortical), timing of onset related to the cardiac arrest, and electroclinical profile. Once considered pathognomonic of invariably poor outcomes [101], a good outcome at hospital discharge can be achieved in patients despite cortical or subcortical myoclonus, although regaining consciousness earlier is more likely in those with subcortical origin [102]. Cortical myoclonus has been recognized to have at least two electroclinical patterns. Elmer pattern 1 (Fig. 5A), characterized by a burst-suppressed background with high amplitude polyspikes in lockstep with myoclonic jerks, has a 100% mortality rate according to a single-center study [103]. In contrast, Elmer pattern 2 (Fig. 5B), characterized by midline-predominant spiky periodic discharges in lockstep with myoclonic jerks with a relatively continuous background, had a 50% survival rate, with all survivors being discharged to home or rehabilitation [103]. Reactivity, long regarded as a harbinger of unfavorable outcomes, has faced scrutiny due to its high rate of false positives [85], and this was confirmed by Liu et al. who found a false positive ratio reaching 25% when a standardized somatosensory stimulus was used to elicit EEG reactivity [104]. This discrepancy is attributed to variations in testing and interpretation standards [105]. An international consensus statement was developed in 2018 by Admiraal et al. generating a stimulus protocol for EEG reactivity testing and the interpretation of EEG reactivity in daily clinical care (Fig. 6). Therefore, EEG has a significant role as a neuroprognostic tool, enabling the prediction of favorable (consciousness and independence) or unfavorable (disorder of consciousness and disabled state) outcomes in a timely dependent manner. EEG is widely used and considered necessary for outcome prediction by most providers caring for cardiac arrest patients [106] and recommended by guidelines [107, 108].

Tables 2 and 3 summarize EEG findings as predictors of good and poor post-arrest outcomes. It is important to note that while no single EEG feature universally guarantees zero false positives or negatives, certain patterns like continuous or generalized periodic discharges on a suppressed EEG background within a specific time frame after the return of spontaneous circulation have been shown to predict poor neurological outcomes with a very low false positive rate. This predictive reliability is particularly noted when these patterns occur within 12–120 h from ROSC, indicating a 100% predictability for poor outcomes under these circumstances [83].

Combining factors like scores [109] or classifications of multiple findings [110, 111] can perform better than individual factors, but they, too, have limitations and lack external validation in large cohorts [109, 110]. This variability in the prediction performance of EEG findings is due to differences in EEG monitoring techniques, timing of findings in correlation to cardiac arrest, timing of outcome assessment, outcome definitions, post-cardiac arrest care practices, neuroprognostication practices that may lead to self-fulfilling prophecies, interrater variability of interpretation of findings [112, 113], definitions of findings, and confounding effects of medications [114].

To address knowledge gaps in both post-cardiac arrest and critically ill populations, it is imperative to navigate nuances and adhere to standardized definitions in EEG monitoring. This approach will improve study interpretation and data pooling from different groups and help identify patients who may derive therapeutic benefits from clinical trials. The TELSTAR trial demonstrated that suppression of all hyperexcitable patterns in cardiac arrest patients, including those not meeting the criteria for electrographic SE or even the ictal-interictal continuum, may not be warranted. However, a trend toward benefit with treatment was seen in patients with unequivocal seizures or evolving patterns [115]. This highlights the need for precise and targeted treatment strategies based on EEG findings to improve patient outcomes.

**Table 2:** Summary of the Performance of EEG Findings as Predictors of Good Outcomes in hypoxic-ischemic brain injury based on EEG timing in relation to the cardiac arrest

	EEG finding ≤ 24 h				EEG finding 24–72 h				EEG finding > 72 h			
	Outcome (m)	Se (%)	Sp (%)	LR	Outcome (m)	Se (%)	Sp (%)	LR	Outcome (m)	Se (%)	Sp (%)	LR
*EEG Predictors of Good Outcome*												
Continuous NOS [116]	D/C	25	100	–	D/C	87.5	77.8	3.9				
Continuous, nearly continuous, or discontinuous [88, 117]^a^					D/C	71.9–100	84.4–96.5	6.1 –20.5	6	77.1	77.1	3.4
Continuous or nearly continuous theta-delta slowing with variability and reactivity [118]	3	85.7–92.3	64.3–77.3	2.6–3.8	3	95.8	67.9	3				
Theta slowing NOS [119]	6	75	90.9	8.2								
Normal or low voltage *without* epileptiform discharges [120–123]	6	56.5–100	87–97.7	7–25	3–6	76.1–100	82.5–87.6	5.2–6.1				
Normal voltage *without* seizures or GPDs [92, 124, 125]	6	51.2–77.8	80.5–97.1	4–19.8	6	62.5–97.9	51–80	2–4.6				
Normal voltage *without* seizures or abundant periodic discharges or spike-wave [86, 125–127]^,b^	6	38.8–84	66.7–100	2.5–3.5	6	45.8	90	4.6	6 m	29.6–77.3	80.1–100	3.9–36.6

Adapted from Sandroni et al. [83] Studies employing amplitude EEG and/or reduced montages were not included

D/C, discharge; EEG, GPD, generalized periodic discharges; electroencephalography; FPR, false positive ratio; h, hours;LR, likelihood ratio; m, months; NOS, not otherwise specified; PDs, periodic discharges; Se, sensitivity; SE, status epilepticus; SIRPIDs, stimulus-induced rhythmic, periodic, or ictal discharges; Sp, specificity

^a^With or without rhythmic or periodic pattern activity

^b^With or without disruption in continuity, with or without reversed anterior–posterior gradient or reactivity

**Table 3:** Summary of the Performance of EEG Findings as Predictors of Poor Outcomes in hypoxic-ischemic brain injury based on EEG timing in relation to the cardiac arrest

	EEG finding < 24 h			EEG finding 24–72 h			EEG finding > 72 h		
	Outcome (m)	Se (%)	FPR%	Outcome (m)	Se (%)	FPR%	Outcome (m)	Se (%)	FPR%
*EEG Predictors of Poor Outcome*									
Isoelectric Background [128]	6	12.9	0	6	7.2	0			
Suppressed Background [116, 118, 126–129]^a^	D/C–6	4.3–63.6	0–14.3	D/C-6	2.1–53.6	0–22.2	6	14.2–25	0
Suppressed background *with* PDs [126, 127, 129]^a^	6	0.4–4	0	6	4–8.7	0	6	2.8–11.8	0
Low voltage Background [126, 127, 129, 130]	6	4.6–32.4	0–9	1–6	4.9–74.4	0–77.8	6	4.3–64.5	0–12.1
Burst suppression (ACNS Defined or synchronous) or attenuation [81, 118, 128–130]	3–6	17.3–41.3	0–4.7	D/C-3	4–30.4	0–23.1	6	1.1–19.7	0–1.5
Burst suppression (Heterogenous or NOS) [116, 119, 129, 131, 132]	D/C-6	9.4–51.5	1.4–50	D/C-6	1.1–55.6	0–50	6	2–3.2	0
Discontinuous Background [128–130]	6	10.8–33.2	8.1–37.6	1–6	6.6–25.6	4. 4.1–13.8	6	26.6–27.5	0–10.3
Unreactive Background [60, 81, 86, 88, 104, 116, 125, 130, 133]^b^	D/C-6	80–97.1	12.5–58.3	D/C-6	50–86.7	0–50	6	88.1	30
Rhythmic /periodic discharges [60, 117, 118, 23, 126–130, 134]	3–6	0.5–42.9	0–2.8	1–6	10.1–50.8	0–33.3	6	5–39.5	0–33.3
Unequivocal seizures or SE [81, 117–119, 126, 127, 129, 130, 132, 135–137]^a^	D/C-6	0.3–36	0–1.8	D/C-6	0.6–33.9	0–4.5	D/C-6	2.1–34.7	0–17.4
SIRPDS [138]	3	10.5	0	3	12.3	2.1			

Adapted from Sandroni et al. [85] Studies employing amplitude EEG and/or reduced montages were not included

D/C, discharge; EEG, GPD, generalized periodic discharges; electroencephalography; FPR, false positive ratio; h, hours; LR, likelihood ratio; m, months; NOS, not otherwise specified; PDs, periodic discharges; Se, sensitivity; SE, status epilepticus; SIRPIDs, stimulus-induced rhythmic, periodic, or ictal discharges; Sp, specificity

^a^Inclusive of studies that used the American Clinical Neurophysiology Society Critical Care EEG terminology [16], other definitions, or no specific definition

^b^Inclusive of studies that used the international consensus on EEG reactivity testing after cardiac arrest [105], other definitions, or no specific definition

### EEG monitoring in traumatic brain injury: strategies and prognostic implications

Traumatic brain injury includes diverse initial injuries such as intracranial hemorrhage, diffuse axonal injury, cerebral contusions, as well as a secondary brain injury due to cerebral edema, traumatic vasospasm, spreading depolarizations, seizures, resulting in a regional mismatch in the supply and demand of blood flow, glucose, oxygen, and pyruvate [46, 139–141]. EEG detection in the acute period following TBI yields important data as early evidence of epileptiform activity on EEG, such as rhythmic or periodic patterns, can serve as markers for subsequent development of post-traumatic epilepsy, which can develop in up to 50% of TBI patients [142] during the first year after injury [143]. Of 34 TBI patients monitored using intracranial EEG, a high incidence of rhythmic and periodic discharges was observed after severe TBI, and this epileptiform activity resulted in a regional metabolic mismatch, or “metabolic crisis”—a state characterized by elevated lactate/pyruvate ratio, decreased extracellular glucose, increased glucose consumption, and/or extracellular brain tissue hypoxia—which may lead to subsequent neuronal injury [46, 140, 141].

CEEG monitoring shortly after admission has been proposed as an add-on marker in patients with moderate to severe TBI, not only to rule out potential nonconvulsive seizures but also to predict long-term clinical outcomes. In a study by Vespa et al., the authors tested the utility of the percentage of alpha variability derived from cEEG monitoring during the early ICU admission of 89 TBI patients to predict their outcome at the time of discharge [144]. Percentages of alpha trends were collected and scored for their variability by a trained technician, and results revealed that a poor percentage of alpha variability score corresponds with a poor 30-day outcome and a high mortality rate. Specifically, within the initial 3 days after injury, a single or average percentage of alpha variability value of 0.1 or lower was highly predictive of poor outcome or death (86%). Similar findings were observed in a more recent study involving 67 severe TBI patients admitted to regular or neurological ICUs [145]. The study results derived that the combination of qEEG and EEG reactivity presented good predictive performance for poor prognosis, such that a predictive model containing relative alpha variability and EEG reactivity was developed and tested, resulting in good discriminative power with an AUC of 0.882 for poor prognosis. However, it is possible that these assessments themselves—or the clinical data they correlate with—influence the clinicians’ prognostication and, therefore, bias these outcomes (self-fulfilling prophecy bias).

Among the current strategies employed in the management of severe TBI cases is optimizing cerebral metabolic demand in the acute post-traumatic period through methods of pharmacological burst suppression or hypothermia. These strategies can reduce secondary neurological insults and improve the chances of recovery [146]. Pharmacologically induced burst suppression, also known as deep anesthesia, is widely utilized in the ICU to decrease the cerebral metabolic rate and ICP in severe TBI patients. Continuous EEG is a useful diagnostic tool for monitoring this induced burst suppression. The goal-directed therapy in TBI patients aims at optimizing the ICP < 20 mmHg, cerebral perfusion pressure > 60 mmHg, and partial pressure of brain tissue oxygen (PbtO2) > 20 mmHg [146]. Additionally, the use of intracranial electrode monitoring in TBI has also been shown to detect cortical spreading depolarization, a phenomenon associated with neurological deterioration and poor outcomes in TBI patients who may be treatment-responsive [44, 147].

Intracranial subdural or intraparenchymal electrodes offer superior sensitivity and localization of seizures [148]. However, their widespread use is limited due to invasiveness and the need for specialized settings. Nonetheless, it's essential for general intensivists to be aware of these techniques to foster discussions about expanding monitoring capabilities and understanding the limitations of scalp EEG. Among ICU patients with coma following an acute brain injury, a small intracranial EEG electrode with a single parenchymal electrode implanted 1–2 cm inside the skull similarly offers enhanced sensitivity for detecting seizures due to direct proximity and reduced signal degradation from suboptimal contact of scalp electrodes, electrical device artifacts, filtering by the skull, or myogenic artifacts that decrease the detection efficiency of scalp EEG [149–151]. Despite recording from a single intracranial location, a single mini-depth intracranial EEG electrode detected EEG-defined seizures or PDs exclusively in 42.9% of adult patients with severe TBI, while surface EEG identified these in only 12 of 21 subjects [140]. This was also confirmed in the study done by Waziri et al. among 14 patients admitted to the ICU with acute brain injury requiring invasive neuromonitoring through the implantation of eight contact mini-depth electrodes [151]. Results showed that intracranial EEG markedly improved signal-to-noise ratio compared with concurrently recorded scalp EEG. Intracranial EEG detected epileptiform findings in 12 of 14 patients (86%), including electrographic seizures (n = 10) and period epileptiform discharges without seizures (n = 2). However, among those with electrographic seizures, scalp EEG never showed an EEG correlate in 6 patients and showed either an intermittent EEG pattern or an intermittent rhythmic delta pattern without a clear evolution in the other four patients. More highly selected albeit heterogeneous cohorts have documented a seizure detection rate exceeding 50% using intracranial EEG [151].

Additionally, intracranial EEG may be performed by placing a cortical electrode strip on the brain’s surface during a craniotomy. This technique allows for the capture of cortical spreading depolarizations from brain injury that are not easily detected by scalp EEG alone [152–156]. While these methods hold potential, their application is still being tested in clinical trials.

### EEG in sepsis-induced brain injury and sepsis-associated encephalopathy

Sepsis is a critical condition that triggers widespread inflammation throughout the body, affecting multiple organ systems, including the brain. This inflammatory response can lead to sepsis-associated encephalopathy, which manifests as various neurological symptoms and is detectable through characteristic EEG changes. Common EEG findings in patients with sepsis include increased theta rhythms, triphasic waves, burst suppression patterns, and periodic epileptiform discharges [157, 158]. These patterns reflect the underlying brain dysfunction and are particularly prevalent in severe cases of systemic infection, affecting up to 70% of ICU patients [159].

In the early mild stages of sepsis-associated encephalopathy, discernible EEG changes begin with theta-range slowing. EEG assists in identifying brain alterations in these early stages before clinical signs of encephalopathy become evident [160]. This can be followed by the emergence of intermittent rhythmic delta activity [161], and as the severity of the condition progresses, persistent rhythmic delta activity may become more prominent. In late severe stages, triphasic waves often appear in association with renal impairment [162], and in the worst cases, EEG patterns may become suppressed entirely, a finding associated with a high mortality rate due to multiorgan failure [161]. In a cohort of 39 sepsis-associated encephalopathy patients, Berisavac et al. showed that delta waves, triphasic waves, and suppression of EEG activity were the most common findings 24 h prior to death [163]. Thus, EEG can help establish the presence of encephalopathy and monitor the course trend of patients with either an improvement or deterioration [161].

### EEG monitoring in aneurysmal subarachnoid hemorrhage management

Among patients who have experienced aSAH, delayed cerebral ischemia (DCI) is one of the most feared complications following surgical management of the culpable aneurysm. EEG abnormalities observed in aSAH patients are closely associated with decreased brain tissue oxygenation and inflammation, which are intermediate findings in the pathway to DCI [44]. This phenomenon is heralded by findings on cEEG: (1) a deterioration in the EEG background (reduced activity in the alpha frequency band, increased activity in the delta frequency band, a decreasing ratio of alpha-to-delta activity, and a reduction of normal variability in the alpha frequency band) [45, 164, 165] as well as (2) new or worsening epileptiform activity [44, 166, 167]. The use of this clinical EEG scoring was confirmed in a prospective study performed among 103 patients admitted with SAH to a single Neurosciences ICU over a 2.5-year study period and underwent cEEG monitoring [5]. Among this population, 52 patients [50.5%] developed DCI, and most of the DCI events were preceded by EEG alarms. Among these EEG alarms, the background deterioration signals (new slowing, decreasing ADR, or decreasing RAV) strongly predicted DCI (63.5% vs. 17.7%; OR 8.11 [3.25–20.2]; *p* < 0.01), and EEG alarms due to new or worsening epileptiform abnormalities even showed a stronger association (63.5% vs. 7.84%, OR 20.4 [6.36–65.5]; *p* < 0.01). Further studies have indicated that while both types of EEG deterioration—background deterioration and new or worsening epileptiform activity—are linked to adverse long-term outcomes, it is possible that background deterioration may improve with clinical management or recovery interventions. Conversely, deterioration attributed to new or worsening epileptiform activity is consistently associated with poorer long-term functional outcomes, either due to lack of treatment or lack of treatment response. Additionally, new or worsening epileptiform activity is linked to elevated brain and blood inflammatory markers levels [168], and brain tissue hypoxia is evident when high-frequency discharges exceed 2 Hz or when frank electrographic seizures occur [44, 169, 170]. A comprehensive approach that integrates both the spatial and temporal features of qEEG data, may allow for the prediction of the occurrence of DCI in alignment with the time of SAH [171].

Patients with SAH are also at risk for deterioration related to cortical spreading depolarizations, which can currently be diagnosed via intracranial EEG with a subdural strip electrode [172]. Claassen and colleagues found that in a group of 48 comatose patients with SAH, 39% exhibited seizure activity on intracortical EEG, whereas only 8% showed seizure activity on scalp EEG [173]. These events occur with high incidence in patients with SAH and may both originate from and exacerbate the mismatch between cerebral metabolic supply and demand, compounding other phenomena such as cerebral vasospasm [174, 175]. A prolonged duration of cortical spreading depolarizations and elevation of the pressure reactivity index can be predictive of DCI. This was shown in a case study describing a patient with aSAH in whom cortical spreading depolarizations and cerebrovascular autoregulation were evaluated using simultaneous electrocorticography and monitoring of the pressure reactivity index after surgical clipping of a ruptured posterior communicating artery aneurysm [176].

## Broader applications of continuous EEG

### EEG in neuroprognostication and rehabilitation of patients in coma and disorders of consciousness

Assessing the level of consciousness in individuals with severe brain injuries is challenging; however, EEG can detect brain activation even without observable behavioral responses to spoken motor commands. A study involving 181 patients with disorders of consciousness due to various etiologies (anoxia, 24%; intracranial hemorrhage, 35%; traumatic brain injury, 24%) used EEG markers such as low-frequency power, EEG complexity, and information exchange to classify the patient’s disorder of conscious grade [177]. These assessments were made at least 24 h after discontinuing sedation to ensure the accuracy of the evaluation with enhanced arousal and cognition. Studies indicate that EEG spectral power, coherence, and entropy effectively differentiate levels of consciousness.

In addition, brain signal diversity, which refers to the variation and complexity of neural signals within a given physiological brain state, has been utilized as a marker indicating the state of consciousness, with a tendency to decrease during unconscious states. Brain signal diversity can be measured by the perturbational complexity index (PCI), diversity of signal complexity, and entropy of high-density EEG when perturbed (evoked) by transcranial magnetic stimulation [178]. This is believed to gauge the brain's current capacity to differentiate and integrate information by measuring the overall complexity of cortical responses to localized perturbations, reflecting both the interconnectedness and the diversity of activity states within the underlying neural system [179]. However, such a system requires significant technical resources as well as the application of high-density electrodes and, thus, has not been widely implemented. In two separate studies involving patients with acute brain injuries, those with EEG evidence of consciousness tended to experience higher rates of good functional recovery [180, 181].

Beyond individual markers, EEG functional connectivity offers insights into the emergence of functional brain networks among patients with disorders of consciousness. Specifically, increased parietal delta and theta activity, along with high frontoparietal theta and alpha coherence, have been identified as early indicators of recovery from the unresponsive wakefulness syndrome (formerly “vegetative state”) with high predictive sensitivity (73%) and specificity (79%) [182]. Computational measures of emerging functional connectivity can also predict a patient’s readiness for liberation from anesthetic coma following treatment for refractory SE. However, these tools are not routinely available due to the absence of quantitative post-processing resources, and functional brain networks are not visibly evident.

Delirium, a form of prevalent acute brain dysfunction in up to 80% of ICU patients, significantly impacts patient outcomes, increasing the risk of mortality, prolonged hospital stays, and persistent cognitive impairment [183–185]. While clinical assessments such as the confusion assessment method–ICU (CAM-ICU) can be helpful for delirium screening, the fluctuating course and pattern of symptoms can pose a diagnostic challenge [186]. cEEG can be informative in these settings; the predominance of low-frequency activity (delta and theta) and reduced high-frequency activity (alpha and beta) serve as an indicator of delirium in critically ill patients [183, 187]. This diagnostic capability is supported by studies like those conducted by Jacobson et al., where EEG differentiated delirium from dementia with a high degree of accuracy (93%) using a brain map scoring system and qEEG metrics like mean posterior dominant frequency, and power ratios in delta, theta, and alpha bands [188]. Similar findings were obtained in a more recent study of 44 subjects examined using several delirium scales, including the confusion assessment method (CAM), and considered positive; their EEG reports revealed the presence of irregular theta slowing with a sensitivity of 93% and a 53% specificity [189].

### Monitoring sedation levels in critically ill patients using EEG

Ensuring an appropriate level of sedation is important to prevent complications in critically ill patients. EEG offers an avenue for tracking sedation levels, enhancing the precision of sedation assessment, and thereby optimizing sedation dosing. For example, a relatively high rate of unintended burst suppression occurs in critically ill and peri-procedural patients managed with IV anesthetics [190–193]. In a study of 26 critically ill adults, monitored with EEG after TBI and SAH, patients were given deep sedation to a Richmond Agitation-Sedation Scale score of − 4 or − 5; most demonstrated a correlation of sedation dosing with one or more EEG indices [194]. An automatic classifier had 84.3% accuracy in discriminating between different sedation doses. Additionally, the time in burst suppression during coma has been associated with the incidence and duration of post-coma delirium [195]. A mediation analysis employing counterfactual statistical analysis showed that burst suppression mediates 10–21% of mortality in a neurocritical care population [196]. The association of propofol use on mortality has no significant direct effect on mortality; the effect of propofol on mortality is entirely mediated through burst suppression.

Automated tools are often used for sedation management, for example, in the setting of neuromuscular blockade. These include the bispectral index monitor (BIS) (Aspect Medical Systems; Natick, MA, USA) [197], or the patient status index (PSI) [117], used for assessing sedation levels via EEG [198]. These indices generally span a scale from 0 (denoting total cortical silence) to 100 (representing an awake state). However, a study by Drover et al. highlighted the efficacy of adjusting propofol administration based on PSI measurements [199]. This approach resulted in swifter emergence and recovery from propofol-alfentanil-nitrous oxide anesthesia, accompanied by a modest reduction in the propofol dosage without any increase in the occurrence of adverse events. While a consensus guideline [200] recommended the use of processed EEG for monitoring sedation in paralyzed or sedated patients without a clinical exam, several challenges were raised including unique signatures of individual sedatives that may actually increase the power of faster frequencies at loss of consciousness—beta activity (e.g., ketamine, nitrous oxide) or alpha activity (e.g., propofol, dexmedetomidine)—or alternatively increase delta power in awake patients (e.g., dexmedetomidine). Other challenges include the potential for epileptiform activity to yield false positives of awake states. This underscores the need for index-based EEG methodologies to be implemented only in the populations and settings in which it was validated.

## Practical considerations and implementation in critical care

### Empowering non-neurophysiologists for EEG utilization

The accurate diagnosis of nonconvulsive SE and nonconvulsive seizures demands specialized neurophysiologic expertise [3], often causing delays in clinical reporting due to the requisite expertise [201]. Quantitative EEG (qEEG) is an assisting tool, which condenses cEEG data into numerical trends interpretable by trained neurointensivists or neurophysiologists. Trends may include spectral power (amplitude of individual frequency bands summed over time), power ratios, variability, rhythmicity, asymmetry, and some tools for spike detection. With training, critical care nurses and other non-neurophysiologists can accurately assess panels displaying real-time qEEG measures such as spectral power, typically reviewing short segments of 15–60 min at a time to identify periods of high-suspicion EEG activity [36, 202, 203]. Following brief qEEG interpretation training 65 ICU nurses achieved 74% sensitivity and 92% specificity in identifying seizures, detecting these seizures 132 min faster than standard neurophysiology practices, although detecting brief events may necessitate zooming in on shorter EEG epochs [203]. Integrating qEEG interpretation training with user-friendly tools empowers non-experts, including critical care nurses, to monitor EEG data for potential seizure activity effectively, promising faster and more accurate seizure detection and improved intervention timing and quality. This approach significantly accelerates the identification of potential electrographic seizures. In one study, ICU nurses employing qEEG detected seizures in 94% of patients with confirmed diagnoses on EEG recordings [204], leading to shorter seizure detection latency compared to conventional practice involving intermittent review by clinical neurophysiologists [203]. Emerging tools utilizing artificial intelligence-powered seizure detection are clinically available, empowering nurses to promptly identify seizures, with some methods even converting EEG data into audible frequency spectra to aid in early detection [205, 206]. However, more complex parameters leveraging quantitative EEG are not yet widely implemented in routine real-time clinical practice at this time, such as functional connectivity (network density, path length, largest, component size, small-worldness, etc.) or stimulus-based measures assessed using ML classifiers.

### Ensuring safety and efficacy during EEG monitoring

Skin breakdown and pressure ulcers are unfortunate but prevalent complications associated with the use of electrodes, particularly in critical care settings where patients may be more vulnerable due to their health conditions. These injuries are prevalent across both pediatric and adult populations, with the incidence of skin breakdown in pediatric and neonatal ICU patients reaching as high as 18.8% [207]. Such injuries often result from prolonged application of metal electrodes on the scalp, contact dermatitis, and the additional stress of abrasions [207–209], compounded by factors like vasopressor use which increases skin vulnerability [210]. A previous retrospective study revealed that the total observed infection rate secondary to cEEG-caused pressure ulcers was 2.4% of all monitored neonatal ICU patients [211]. To combat these risks, the ASET Skin Safety Task Force has developed guidelines for EEG procedures, focusing on techniques over products, considering factors affecting skin response, and recommending additional precautions for vulnerable populations, including patients undergoing long-term EEG monitoring [212]. Thus, to minimize these injuries, intervention at the electrode level is necessary, including utilizing less abrasive skin preparation solutions, disposable electrodes, lack of tight head wraps, and daily skin inspection with a small movement of an electrode’s placement when early-stage skin irritation is evident. These practices have successfully reduced EEG electrode-related skin injury among vulnerable patients [209, 211, 213].

### Facilitating patient mobility during continuous EEG monitoring

Early mobilization in the ICU has garnered significant attention. For example, studies have shown that early mobilization can improve functional capacity, muscle strength, walking distance, and overall quality of life for patients [214]. International practice guidelines advocate early mobilization as a safe and feasible practice in the ICU setting [215]. Physical therapy and mobility exercises are important for maintaining muscle strength and preventing complications such as thrombosis [216].

Patients undergoing cEEG monitoring can also benefit from mobility interventions. Many cEEG electrode systems are designed to allow intermittent disconnection, facilitating patient ambulation and movement between different positions, such as transferring from the bed to a chair. Several effective strategies have been adopted to mobilize ICU patients during cEEG monitoring, including: briefly disconnecting patients who are being repositioned to prevent traction on electrodes; in-bed exercises such as leg lifts, ankle pumps, and knee bends; in-bed mobility using pillows to support the head and prevent movement of the EEG electrodes; and taping electrodes to the head during assisted ambulation. This close interdisciplinary collaboration can maximize mobility while avoiding the need to remove and reapply electrodes frequently.

## Summary

Continuous EEG monitoring is a valuable tool in the detection, classification, and management of seizures in critically ill patients, offering improved seizure detection and localization compared to brief EEG. Early detection of seizures, particularly nonconvulsive seizures, is important given the risk of this activity resulting in secondary brain injury. Quantitative EEG measures empower non-neurophysiologists, such as critical care nurses, to accurately assess EEG patterns and promptly alert clinicians to potential seizure activity. This streamlined approach enhances the timeliness of interventions and augments patient care. Intracranial EEG provides enhanced sensitivity in detecting seizures among patients with acute brain injury. Detecting spreading depolarizations through intracranial EEG adds a new dimension to understanding brain injury and may guide targeted therapeutic interventions.

The utility of cEEG extends beyond epilepsy to other aspects of critical care, such as identifying and monitoring encephalopathy, assessing anesthesia and sedation levels, and predicting outcomes following traumatic injuries and cardiac arrest. Additionally, cEEG aids in diagnosing and managing sepsis-associated encephalopathy, which often presents with subtle EEG changes indicative of neurological dysfunction.

Despite these diverse opportunities, implementing cEEG monitoring is challenging. Electrode-related skin injuries and pressure ulcers can pose risks, particularly in vulnerable populations. Effective strategies, including proper skin preparation, electrode repositioning, and regular skin inspections, limit the occurrence of these complications. Additionally, facilitating early mobilization in ICU patients undergoing continuous EEG monitoring requires a multidisciplinary approach that ensures patient safety and recording integrity.

In conclusion, cEEG monitoring is a cornerstone in critical care management, offering real-time insights into brain function and aiding clinical decision-making across neurological conditions. From guiding treatment strategies to enhancing prognostication accuracy, continuous EEG empowers healthcare providers to optimize patient care and outcomes in a complex and rapidly evolving landscape.

## Data Availability

Not applicable.

## Abbreviations

ACNS: American Clinical Neurophysiology Society
aSAH: Aneurysmal subarachnoid hemorrhage
ASM: Antiseizure medication
BIPDs: Bilateral independent periodic discharges
BIRDs: Brief potentially ictal rhythmic discharges
BIS: Bispectral index monitor
CAM-ICU: Confusion assessment method-intensive care unit
cEEG: Continuous electroencephalography
DCI: Delayed cerebral ischemia
ED: Emergency department
EEG: Electroencephalography
ESETT: Established status epilepticus treatment trial
GPDs: Generalized periodic discharges
GRDA: Generalized rhythmic delta activity
ICP: Intracranial pressure
ICU: Intensive care unit
IIC: Ictal-interictal continuum
LPDs: Lateralized periodic discharges
LRDA: Lateralized rhythmic delta activity
MRI: Magnetic resonance imaging
PbtO2: Partial pressure of brain tissue oxygen
PD: Periodic discharge
PSI: Patient status index
qEEG: Quantitative electroencephalography
RDA: Rhythmic delta activity
RPP: Rhythmic or periodic pattern
SAH: Subarachnoid hemorrhage
SE: Status epilepticus
SIRPIDs: Stimulus induced rhythmic periodic intermittent discharges
TBI: Traumatic brain injury
